# Supplementary material for: A walk in the maze: variation in Late Jurassic tridactyl dinosaur tracks from the Swiss Jura Mountains (NW Switzerland)
Source: PeerJ. 2018 Apr 2;6:e4579. doi: 10.7717/peerj.4579 (PMC5885975; doi:10.7717/peerj.4579)
Supplement: Supplemental Information 1 — Those with preservation grade 0–0.5 are not included in the figshare file. The tracks where the variation along the trackway has been analysed are in red. [file peerj-06-4579-s001.docx]

| Slab | Trackway/Track | Grade | Maximum  depth (mm) | Classification |
| --- | --- | --- | --- | --- |
| BEB011-175 | BEB500-E1 | 1,5 | 10,5 | robust |
| BEB012-r1 | BEB500-T10-L6 | 1 | 6,2 | grallatorid |
| BEB012-r2 | BEB500-T120-L5 | 1 | 4,2 | robust |
| BEB012-r2 | BEB500-T120-L6 | 0 | none | robust |
| BEB012-r2 | BEB500-T120-R5 | 2 | 6,1 | robust |
| BEB012-r2 | BEB500-T120-R6 | 2 | 10 | robust |
| BEB012-r1 | BEB500-T16-L4 | 1 | 4,6 | grallatorid |
| BEB012-r1 | BEB500-T16-R3 | 2,5 | 5,6 | grallatorid |
| BEB012-r1 | BEB500-T16-R4 | 0,5 | 5,7 | grallatorid |
| BEB012-r10 | BEB500-T17-L8 | 1 | 5,5 | grallatorid |
| BEB012-r10 | BEB500-T17-L9 | 1,5 | 4,2 | grallatorid |
| BEB012-r5 | BEB500-T17-R20 | 1,5 | 7 | grallatorid |
| BEB012-r10 | BEB500-T17-R8 | 2 | 4,2 | grallatorid |
| LASER-SCAN | BEB-500-T19-R8 | 1,5 | 5,1 | grallatorid |
| BEB011-r60 | BEB500-T26-R5 | 2 | 4,6 | grallatorid |
| LASER-SCAN | BEB-500-T37-L11 | 2 | 6,5 | grallatorid |
| LASER-SCAN | BEB-500-T54-R11 | 2 | 3,1 | grallatorid |
| LASER-SCAN | BEB-500-T57-L3 | 1,5 | 4,1 | grallatorid |
| BEB012-r9 | BEB500-T58-L21 | 1 | 5,1 | grallatorid |
| BEB012-r9 | BEB500-T58-L22 | 1,5 | 3,9 | grallatorid |
| BEB012-r9 | BEB500-T58-L23 | 1 | 6,2 | grallatorid |
| BEB012-r9 | BEB500-T58-R20 | 1,5 | 5,2 | grallatorid |
| BEB012-r9 | BEB500-T58-R21 | 1,5 | 4,5 | grallatorid |
| BEB012-r9 | BEB500-T58-R22 | 0,5 | none | grallatorid |
| LASER-SCAN | BEB-500-T64-L4 | 1 | 6,2 | grallatorid |
| BEB012-r5 | BEB500-T73-L3 | 1,5 | 5,1 | grallatorid |
| BEB011-r60  / BEB011-161 | BEB500-T73-L5 | 2 | 6,9 | grallatorid |
| BEB011-r60 | BEB500-T73-R4 | 1 | 5,6 | grallatorid |
| BEB011-r60 | BEB500-T73-R5 | 1,5 | 4,9 | grallatorid |
| BEB011-125 | BEB500-T75-R12 | 1,5 | 3,3 | robust |
| LASER-SCAN | BEB-500-T75-R15 | 1,5 | 3,3 | robust |
| BEB012-r1 | BEB500-T76-R2 | 1 | 5,7 | grallatorid |
| BEB012-r7 | BEB500-T78-L5 | 1 | 5,7 | grallatorid |
| BEB012-r6 | BEB500-T78-R3 | 1 | 6,1 | grallatorid |
| LASER-SCAN | BEB-500-T82-R9 | 1,5 | 4,8 | grallatorid |
| LASER-SCAN | BEB-500-T82-R14 | 1,5 | 6,7 | grallatorid |
| LASER-SCAN | BEB-500-T93-R6 | 1 | 7,8 | grallatorid |
| LASER-SCAN | BEB-500-T93-L5 | 1,5 | 4 | grallatorid |
| BSY009-38 | BSY1020-E2 | 2,5 | 7,2 | grallatorid |
| BSY008-319 | BSY1040-T5-R1 | 1,5 | 3,2 | grallatorid |
| BSY008-200 | BSY1040-T6-R4 | 1 | 6,1 | grallatorid |
| BSY008-145 | BSY1050-T1-L11 | 1 | 12 | grallatorid |
| CHV004-22 | CHV1000-E3 | 1,5 | 6,8 | grallatorid |
| Slab | Trackway/Track | Grade | Maximum  depth (mm) | Classification |
| CHV004-22 | CHV1000-E4 | 2 | 9 | grallatorid |
| CRO006-r44 | CRO500-T10-R9 | 1,5 | 3,4 | grallatorid |
| CRO006-r44 | CRO500-T10-L10 | 2 | 4,9 | grallatorid |
| CRO006-r50 | CRO500-T10-L17 | 1,5 | 3,8 | grallatorid |
| CRO006-r50 | CRO500-T10-L18 | 0,5 | none | grallatorid |
| CRO006-r50 | CRO500-T10-L20 | 1 | 4,2 | grallatorid |
| CRO006-r35 | CRO500-T10-L3 | 0 | none | grallatorid |
| CRO006-r35 | CRO500-T10-L4 | 0,5 | 4,8 | grallatorid |
| CRO006-r35 | CRO500-T10-L5 | 0 | 5,7 | grallatorid |
| CRO006-r50 | CRO500-T10-R17 | 0,5 | none | grallatorid |
| CRO006-r50 | CRO500-T10-R18 | 0,5 | none | grallatorid |
| CRO006-r50 | CRO500-T10-R19 | 1,5 | 4,8 | grallatorid |
| CRO006-r50 | CRO500-T10-R20 | 0 | none | grallatorid |
| CRO006-r35 | CRO500-T10-R3 | 1,5 | 3,1 | grallatorid |
| CRO006-r35 | CRO500-T10-R4 | 1,5 | 4,3 | grallatorid |
| CRO006-r44 | CRO500-T14-R5 | 1 | 8,7 | robust |
| CRO007-r24 | CRO500-T30BIS-L4 | 0 | none | grallatorid |
| CRO007-r24 | CRO500-T30BIS-L5 | 2 | 5,3 | grallatorid |
| CRO007-r24 | CRO500-T30BIS-L6 | 1 | 5,9 | grallatorid |
| CRO007-r24 | CRO500-T30BIS-R4 | 1 | 7,4 | grallatorid |
| CRO007-r24 | CRO500-T30BIS-R5 | 1,5 | 10 | grallatorid |
| SCR011-592 | SCR1055-T2-L2 | 2 | 8 | grallatorid |
| SCR011-592 | SCR1055-T3-L2 | 2 | 9,1 | grallatorid |
| TCH006-1086 | TCH1055-E53 | 2 | 5,8 | grallatorid |
| TCH006-1086 | TCH1055-E54 | 1 | 4,9 | grallatorid |
| TCH006-1100 | TCH1055-T2-L1 | 2 | 5,1 | grallatorid |
| TCH006-1301 | TCH1055-T2-R1 | 2,5 | 7,6 | grallatorid |
| TCH006-1100 | TCH1055-T3-R1 | 1,5 | 5 | grallatorid |
| TCH0006-451 | TCH1060-E58 | 2,5 | 5,7 | grallatorid |
| TCH006-419 | TCH1060-T7-R1 | 1 | 6,2 | grallatorid |
| TCH006-419 | TCH1060-T10-L3 | 1 | 9,1 | grallatorid |
| TCH006-59 | TCH1065-E3 | 2 | 9,1 | grallatorid |
| TCH006-64 | TCH1065-E28 | 1,5 | 11,7 | robust |
| TCH006-64 | TCH1065-E99 | 1,5 | 8 | grallatorid |
| TCH006-92 | TCH1065-E124 | 2 | 6,9 | robust |
| TCH006-83 | TCH1065-E176 | 1 | 15 | grallatorid |
| TCH006-83 | TCH1065-E177 | 2 | 6,9 | grallatorid |
| TCH006-403 | TCH1065-E180 | 1,5 | 10,6 | grallatorid |
| TCH006-409 | TCH1065-E188 | 2 | 5,9 | robust |
| TCH006-62 | TCH1065-T3-R2 | 1,5 | 7,1 | robust |
| TCH006-62 | TCH1065-T15-L1 | 0,5 | 6,8 | robust |
| TCH006-62 | TCH1065-T15-R1 | 2 | 8,3 | robust |
| Slab | Trackway/Track | Grade | Maximum  depth (mm) | Classification |
| TCH006-78 /  TCH005-r136 | TCH1065-T21-R1 | 2 | 12,1 | robust |
| TCH006-92 | TCH1065-T16-R2 | 0,5 | none | none |
| TCH006-405 | TCH1065-T25-L2 | 2 | 10,2 | grallatorid |
| TCH006-403 | TCH1065-T25-R2 | 1 | 12,9 | grallatorid |
| TCH006-10 | TCH1069-T1-R2 | 2 | 5,7 | grallatorid |
| TCH006-6 | TCH1069-T2-L2 | 1,5 | 9,6 | robust |
| TCH006-16 | TCH1069-T2-R3 | 1 | 7,8 | robust |
| TCH006-6 | TCH1069-T4-L2 | 1,5 | 4,2 | grallatorid |

Supplemental information Table S1: List of the specimens analysed, their quality of preservation (preservation grade) and the maximum depth. Those with preservation grade 0-0.5 are not included in the figshare file. The tracks where the variation along the trackway has been analysed are in red (see also Table 2).
