# Supplementary figures and images for: A walk in the maze: variation in Late Jurassic tridactyl dinosaur tracks from the Swiss Jura Mountains (NW Switzerland)
Source: PeerJ. 2018 Apr 2;6:e4579. doi: 10.7717/peerj.4579 (PMC5885975; doi:10.7717/peerj.4579)

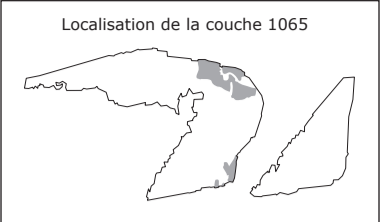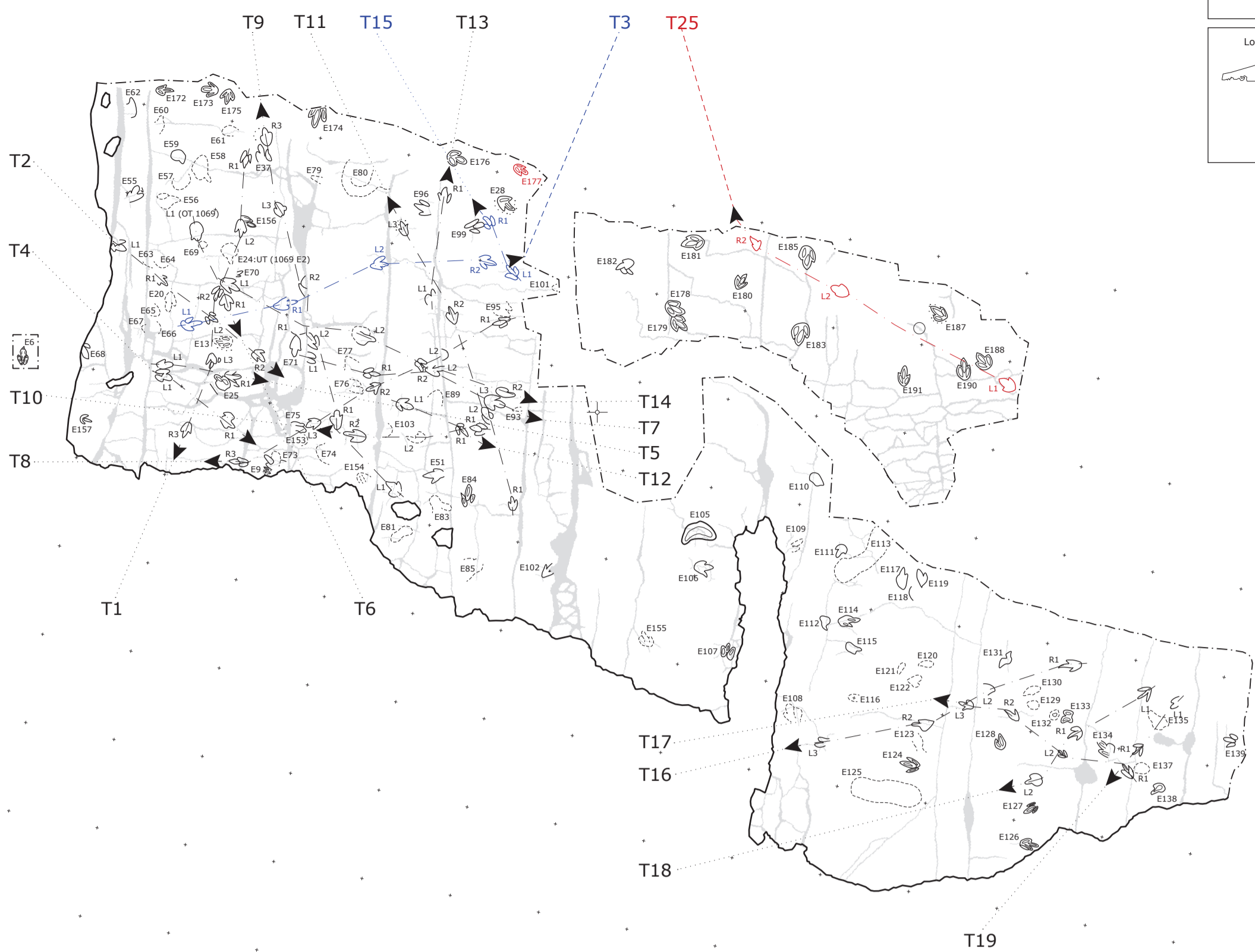

568610/250420

15/45  
568610.088/250418.171  
10.832°

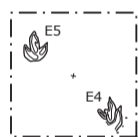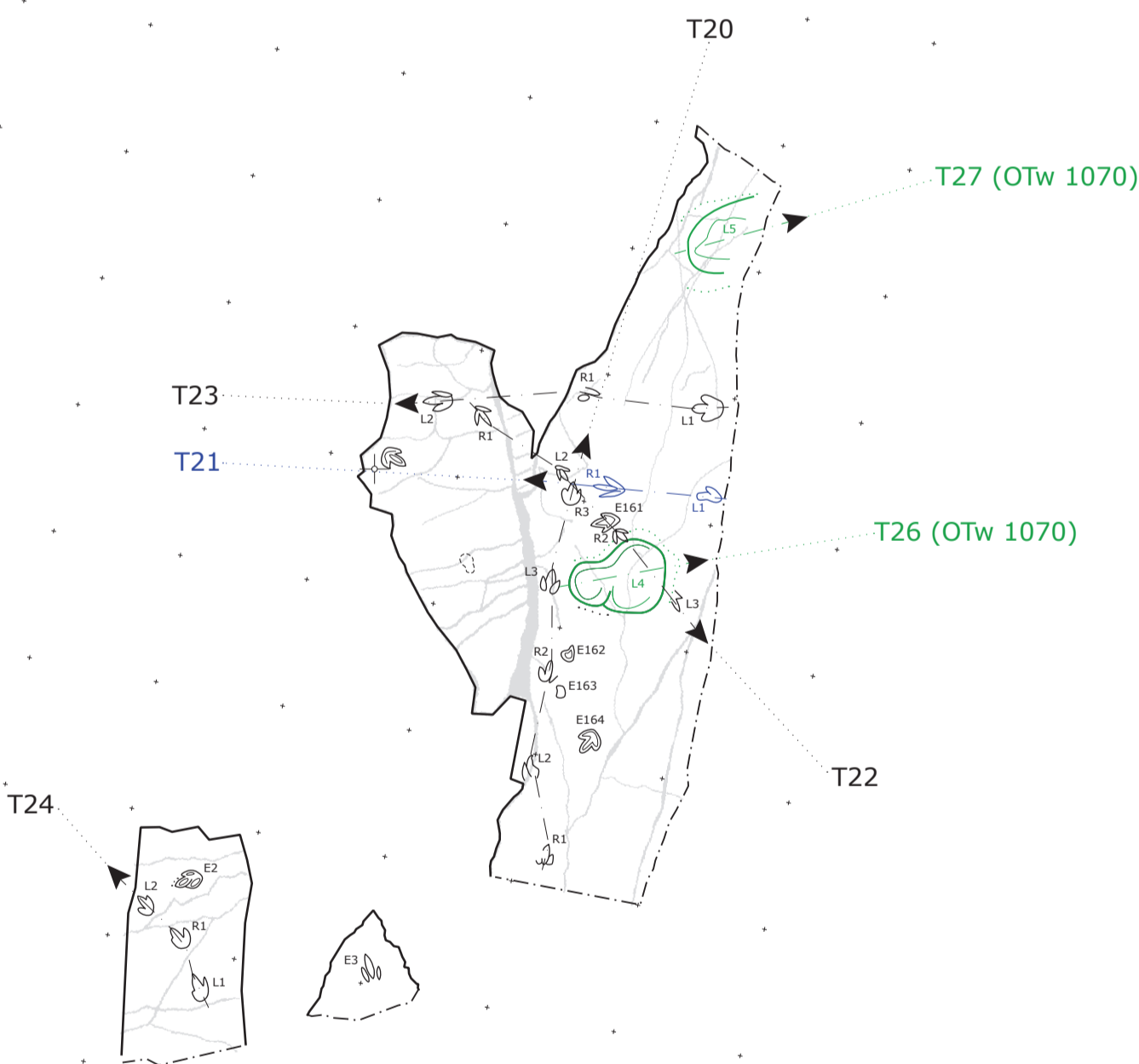

Supplement: Supplemental Information 3 — In red (gracile) and blue (robust) the minute to medium-sized tridactyl tracks and in green, the larger morphtoype (Jurabrontes curtedulensis see Marty et al., 2017). Source credit: OCC-SAP, Canton Jura. [file peerj-06-4579-s003.pdf]

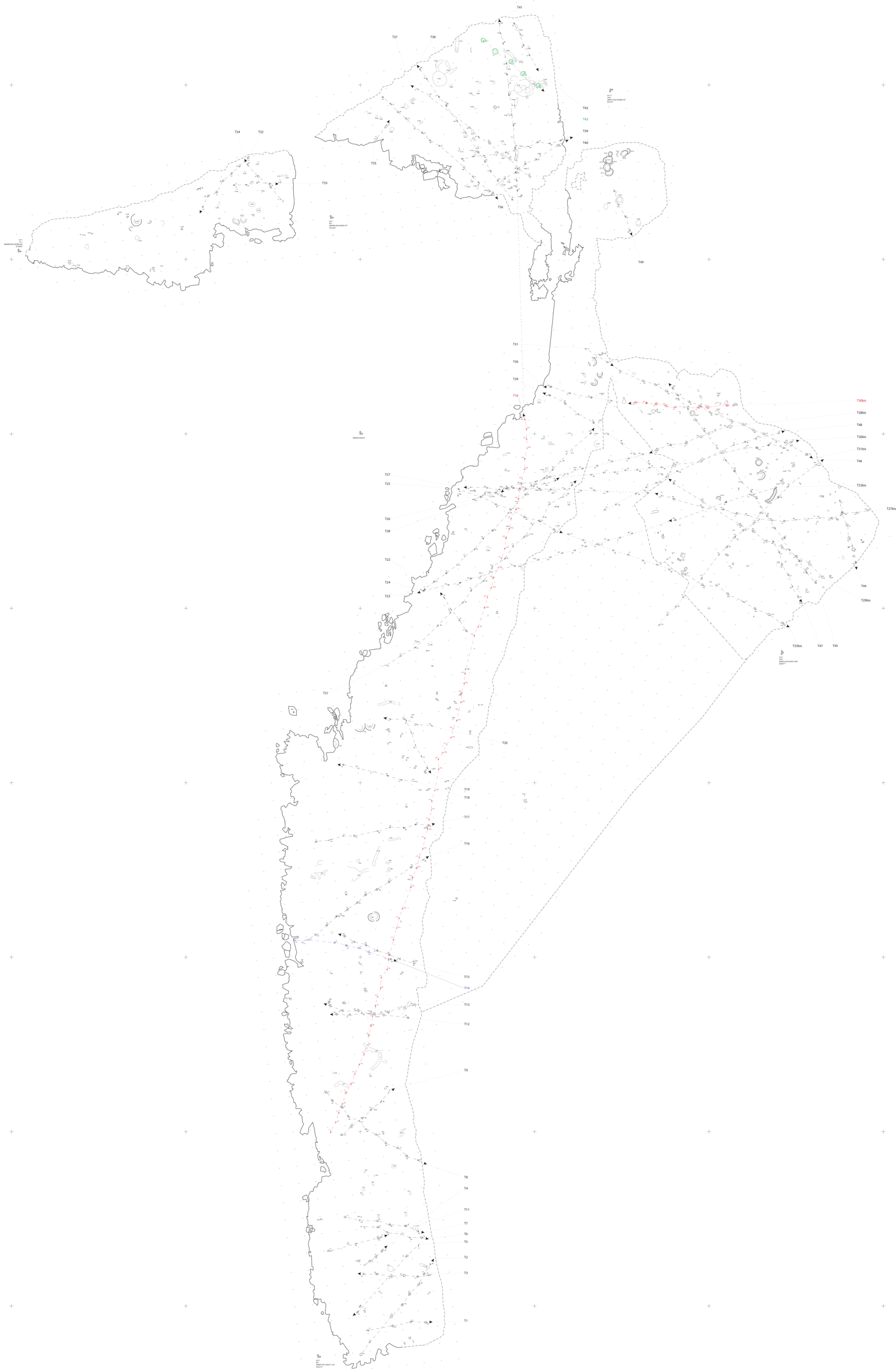

Supplement: Supplemental Information 4 — In red (gracile) and blue (robust) the minute to medium-sized tridactyl tracks and in green, the larger morphtoype (Morphotype II). Source credit: OCC-SAP, Canton Jura. [file peerj-06-4579-s004.pdf]
