# Supplementary material for: A walk in the maze: variation in Late Jurassic tridactyl dinosaur tracks from the Swiss Jura Mountains (NW Switzerland)
Source: PeerJ. 2018 Apr 2;6:e4579. doi: 10.7717/peerj.4579 (PMC5885975; doi:10.7717/peerj.4579)

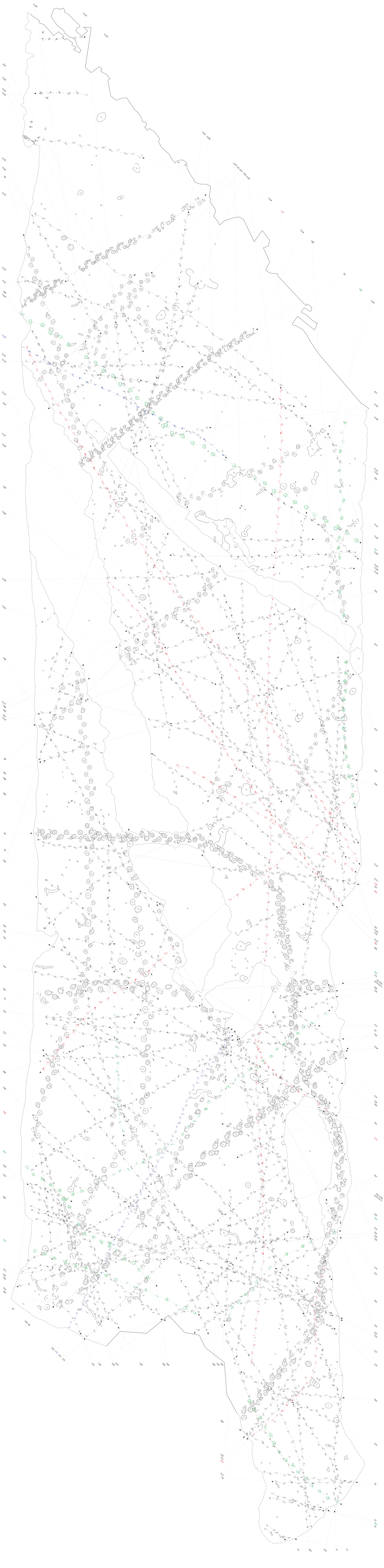

Courtedoux-Béchat Bovais

Plan index des moulages
Couche 500
Secteur 1

0 1:50 1m

orienté selon les coordonnées locales

Localisation de la couche 500

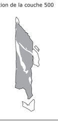

Supplement: Supplemental Information 2 — In red (gracile) and blue (robust) the minute to medium-sized tridactyl tracks and in green, the larger morphtoype (Morphotype II). Source credit: OCC-SAP, Canton Jura. [file peerj-06-4579-s002.pdf]
